# Crystal structure of (3S*,4R*)-4-fluoro-3-(4-meth­oxy­phen­yl)-1-oxo-2-phenyl-1,2,3,4-tetra­hydro­iso­quinoline-4-carb­oxy­lic acid

**DOI:** 10.1107/S2056989017007186

**Published:** 2017-05-23

**Authors:** Anna Lehmann, Lisa Lechner, Krzysztof Radacki, Holger Braunschweig, Ulrike Holzgrabe

**Affiliations:** a Institute of Pharmacy and Food Chemistry, University of Wuerzburg, Am Hubland, 97074 Wuerzburg, Germany; b Institute of Inorganic Chemistry, University of Wuerzburg, Am Hubland, 97074 Wuerzburg, Germany

**Keywords:** crystal structure, *cis* conformation, fluorination, 1-oxo­tetra­hydro­iso­quinolinon-4-carb­oxy­lic acid, hydrogen bonding

## Abstract

The title compound, C_23_H_18_FNO_4_, crystallized as a racemate. It exhibits a *cis* conformation with respect to the F atom and the methine H atom. The piperidine ring has a screw-boat conformation. The meth­oxy­phenyl ring and the phenyl ring are inclined to the mean plane of the iso­quinoline ring system by 89.85 (4) and 46.62 (5)°, respectively, and by 78.15 (5)° to one another. In the crystal, mol­ecules are linked by an O—H⋯O hydrogen bond forming chains propagating along the *a*-axis direction. The chains are linked by C—H⋯F hydrogen bonds, forming layers lying parallel to the *ab* plane.

## Abstract 2

The title compound exhibits a *cis* conformation with respect to the F atom and the methine H atom. It crystallized from a racemic mixture as a pure diastereomer, hence the unit-cell contains both the 3*S*,4*R*- and 3*R*,4*S*-enanti­omers.

## Chemical context   

Several decades ago, Cushman *et al.* (1977 ▸) described a general synthesis of 4-carb­oxy-3,4-di­hydro­isoquinolin-1(*2H*)-ones by a condensation reaction of various aldimines with homophthalic anhydride. In most cases, a mixture of *trans* and *cis* diastereomers was obtained. As the *trans* isomer is the thermodynamically more stable product, it was possible to epimerize the *cis* compound completely to the more stable isoform. Accordingly, Haimova *et al.* (1977 ▸) reported the isolation of the pure thermodynamic product after the treatment of the reaction mixture with 10% NaOH solution.

**Figure 1:**
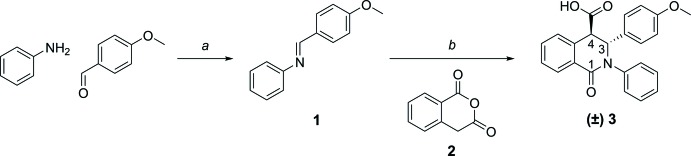
Synthesis scheme to obtain the *trans*-isomer (±) **3**. Reagents and conditions: (*a*) EtOH, r.t., 3 h; (*b*) homophthalic anhydride (**2**), conc. HOAc, 393 K, 5 h; EtOH, 8 *M* NaOH, r.t., 24 h.

Combined synthesis conditions resulted in isolation of stereopure *trans* compound (±) **3** (Fig. 1 ▸). First of all, the imine derivative **1** was synthesized by condensation of 4-meth­oxy­benzaldehyde and aniline. Conversion of homophthalic anhydride **2** with **1** in conc. HOAc gave a diastereomeric *cis*/*trans* mixture, which was completely converted to the pure *trans* enanti­omers by treatment with 8 *M* NaOH solution. The *cis*/*trans* isomers can be differentiated by the proton-coupling constants *J_AB_* between H-3 and H-4, being *J_AB_* 1.5 Hz for the *trans* compounds and *J_AB_* 6.0 Hz for the *cis* isomers.

**Figure 2:**
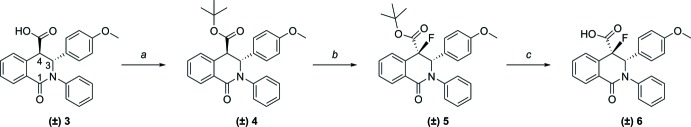
Synthesis scheme to obtain the fluorinated *cis*-enanti­omers (±) **6**. Reagents and conditions: (*a*) absolute THF, DMAP, di-*tert*-butyl dicarbonate, r.t., 24 h; (*b*) absolute THF, LiHMDS, NFSI, 201 K–r.t., 42 h; (*c*) CH_3_CN, 85% (*w*/*w*) H_3_PO_4_, 323 K, 4 d.

To prevent epimerization during subsequent synthesis steps, *e.g.* an amide formation, the isosteric substitution of the acidic proton H-4 by a fluorine atom was investigated (Fig. 2 ▸). First, the carb­oxy­lic acid (±) **3** was protected by *tert*-butyl ester to obtain the ester (±) **4** (Takeda *et al.*, 1994 ▸). Fluorination to (±) **5** was achieved by deprotonation with lithiumbis(tri­methyl­sil­yl)amide (LiHMDS) and addition of *N-*fluoro­benzene­sulfonimide (NFSI) (Differding & Ofner, 1991 ▸; Davis *et al.*, 1995 ▸). Finally, the fluorinated product was deprotected using mild conditions (Li *et al.*, 2006 ▸) to obtain the pure diastereomer (±) **6**.
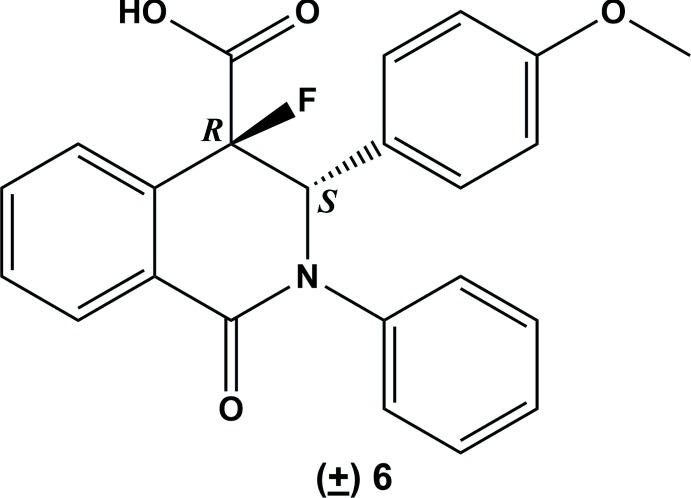


## Structural commentary   

**Figure 3:**
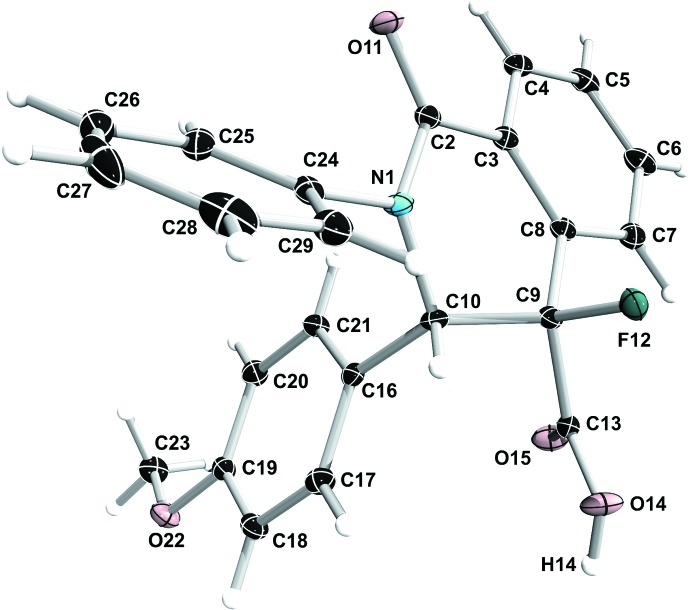
The mol­ecular structure of compound (±) **6**, with atom labelling and 50% probability displacement ellipsoids.

Compound (±) **6** exhibits a *cis*-conformation with respect to the fluorine atom F12 and the H atom H10, as shown in Fig. 3 ▸. The piperidine ring (N1/C2/C3/C8–C10) has a screw-boat conformation [puckering amplitude *Q* = 0.3812 (11) Å, θ = 64.50 (17)°, φ = 279.15 (18)°]. The meth­oxy­phenyl ring (C16–C21) and the phenyl ring (C24–C29) are inclined to the mean plane of the iso­quinoline ring system (N1/C1–C10) by 89.85 (4) and 46.62 (5)°, respectively, and by 78.15 (5)° to one another.

## Supra­molecular features   

**Figure 4:**
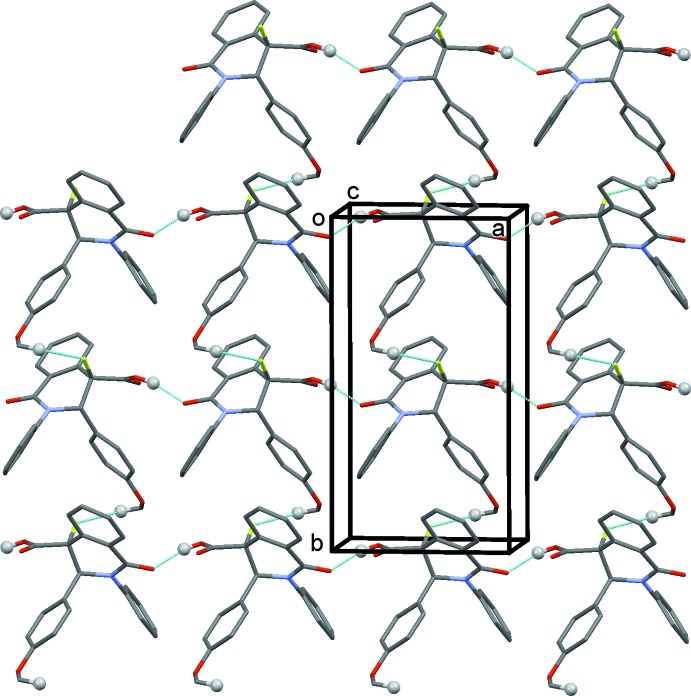
A view along the *c* axis of the crystal packing of compound (±) **6**, with hydrogen bonds drawn as dashed lines (see Table 1 ▸). For clarity, only H atoms H14 and H23*C* (grey balls) have been included.

In the crystal, mol­ecules are linked by an O—H⋯O hydrogen bond, between the carb­oxy­lic OH group (OH14) and amide oxygen atom (O11), forming chains propagating along the *a*-axis direction (Fig. 4 ▸ and Table 1 ▸). The chains are linked by C—H⋯F hydrogen bonds, forming layers parallel to the *ab* plane (Fig. 4 ▸ and Table 1 ▸). Individual chains are homo-chiral, with adjacent molecules related by translation only. It is interesting that carboxylate inversion dimers are not observed. It is supposed that the formation of such dimers is hindered by the quite strong F⋯ H interactions, causing a fixed arrangement between the chain layers.

## Synthesis and crystallization   

The synthesis of the title compound, (±) **6**, is outlined in Figs. 1 ▸ and 2 ▸.

**1-(4-Meth­oxy­phen­yl)-** *N* **-phenyl­methanimine (1)**: Synthesized according to the procedure reported by Torregrosa *et al.* (2005 ▸). The imine was prepared by condensation of 4-meth­oxy­benzaldehyde (5.00 g, 36.7 mmol) and aniline (3.40 ml, 36.7 mmol) in EtOH (20 ml) at room temperature to obtain colourless crystals (yield 7.20 g, 34.0 mmol, 92%). The NMR spectra and melting point corresponds to reported data (Torregrosa *et al.*, 2005 ▸).

***trans*** **-3-(4-Meth­oxy­phen­yl)-1-oxo-2-phenyl-1,2,3,4-tetra­hydro­iso­quinoline-4-carb­oxy­lic acid, (**±**) 3**: Synthesized according to the procedure reported by Guy *et al.* (2013 ▸). Homophthalic anhydride (3.50 g, 21.5 mmol) was dissolved in conc. HOAc, **1** (6.00 g, 28.4 mmol) was added and the reaction mixture stirred for 4 h at 393 K. Afterwards, the mixture was adjusted to neutral pH value with NaOH solution and extracted with CHCl_3_, the organic phase dried over Na_2_SO_4_ and concentrated *in vacuo*. The crude product was purified by silica gel chromatography (CHCl_3_/EtOH/FA 10/0.3/0.1) to isolate mixture of *cis*/*trans*-diastereomers. The solid was dissolved in EtOH (10 ml), 8 *M* NaOH solution (2.30 ml) was added and the reaction mixture stirred for 24 h at room temperature. After adjusting the pH value to acidic conditions, the mixture was extracted with CHCl_3_, dried over Na_2_SO_4_ and concentrated *in vacuo* to obtain a racemic mixture of *trans*-enanti­omers as a colourless amorphous solid (yield 6.50 g, 17.3 mmol, 77%; m.p. 443–444 K). ^1^H NMR (CDCl_3_, 400 MHz): δ 8.27–8.22 (*m*, 1H), 7.49–7.44 (*m*, 2H), 7.27–7.16 (*m*, 6H), 7.05–7.01 (*m*, 2H), 6.75–6.72 (*m*, 2H), 5.52 (*s*, 1H), 3.97 (*d*, *J* = 1.4 Hz, 1H), 3.72 (*s*, 3H). ^13^C NMR (CDCl_3_, 100 MHz): δ 174.5, 163.7, 159.4, 142.2, 132.7, 132.2, 130.9, 129.6, 129.5, 129.1, 128.8, 128.6, 127.7 (2C), 127.3, 126.9, 114.3 (2C), 64.4, 55.3, 51.6. IR 1723, 1602, 1510, 1491, 1462, 1247, 1172, 1027, 828, 730, 693, 628 cm^−1^. ESI–MS: *m*/*z* 374.2 [*M* + H^+^].

***tert*** **-Butyl-** ***trans*** **-3-(4-meth­oxy­phen­yl)-1-οxo-2-phenyl-1,2,3,4-tetra­hydro­iso­quinoline-4-carboxyl­ate, (**±**) 4:** 2.50 g of **3** (6.70 mmol) were dissolved in abs. THF (70 ml). After the addition of di-*tert*-butyl­dicarbonate (1.40 ml, 6.00 mmol) and DMAP (81.5 mg, 0.70 mmol) the reaction mixture stirred for 24 h at room temperature. Afterwards the reaction was quenched with water (100 ml), extracted with CHCl_3_, dried over Na_2_SO_4_ and concentrated *in vacuo*. The crude product was purified by MPLC (petroleum ether/EtOAc 1/0 to 0/1) to isolate **4** as a colourless amorphous solid (yield 1.60 g, 3.70 mmol, 55%; m.p. 421–422 K). ^1^H NMR (CDCl_3_, 400 MHz): δ 8.25–8.21 (*m*, 1H), 7.45–7.41 (*m*, 2H), 7.33–7.32 (*m*, 4H), 7.24–7.19 (*m*, 1H), 7.17–7.15 (*m*, 1H), 7.08–7.05 (*m*, 2H), 6.75–6.71 (*m*, 2H), 5.54 (*d*, *J* = 1.4 Hz, 1H), 3.89 (*d*, *J* = 1.7 Hz, 1H), 3.71 (*s*, 3H), 1.38 (*s*, 9H). ^13^C NMR (CDCl_3_, 100 MHz): δ 169.9, 163.5, 159.2, 142.6, 133.4, 132.3, 131.6, 129.6, 129.4, 129.0, 128.4, 128.3, 127.7 (2C), 126.9, 126.6, 114.2 (2C), 82.5, 64.7, 55.3, 53.2, 28.0. IR 2975, 1730, 1661, 1510, 1399, 1300, 1244, 1139, 1028, 826 cm^−1^. ESI–MS: *m*/*z* 430.1 [*M* + H^+^].

***tert*** **-Butyl-** ***cis*** **-4-fluoro-3-(4-μeth­oxy­phen­yl)-1-oxo-2-phen­yl-1,2,3,4-tetra­hydro­iso­quinoline-4-carboxyl­ate, (**±**) 5:** 500 mg of compound **4** (1.20 mmol) were dissolved in abs. THF (38 ml) under an argon atmosphere and cooled to 301 K. After the addition of 1 *M* LiHMDS solution (1.40 ml, 1.40 mmol), the mixture was stirred for 1 h while cooling. Afterwards, NFSI (511 mg, 1.60 mmol) was added and the mixture stirred for a further 30 min at 301 K and then 40 h at room temperature. The reaction mixture was extracted with CHCl_3_, dried over Na_2_SO_4_ and concentrated *in vacuo*. The crude product was purified by MPLC (petroleum ether/EtOAc 1/0 to 0/1) to isolate **(**±**) 5** as colourless crystals (yield 320 mg, 0.70 mmol, 62%; m.p. 452–453 K). ^1^H NMR (CDCl_3_, 400 MHz): δ 8.41–8.39 (*m*, 1H), 7.69–7.60 (*m*, 2H), 7.51–7.48 (*m*, 1H), 7.34–7.29 (*m*, 2H), 7.27–7.23 (*m*, 1H), 7.11–7.07 (*m*, 2H), 6.92–6.88 (*m*, 2H), 6.72–6.68 (*m*, 2H), 5.21 (*d*, *J* = 15.7 Hz, 1H), 3.73 (*s*, 3H), 1.26 (*s*, 9H). ^13^C NMR (CDCl_3_, 100 MHz): δ 165.8 (*d*, *J*_CF_ = 26.8 Hz), 162.0 (*d*, *J*_CF_ = 1.3 Hz), 160.1, 141.3, 132.6 (*d*, *J*_CF_ = 2.9 Hz), 132.3, 130.9 (*d*, *J*_CF_ = 3.9 Hz), 130.3 (*d*, *J*_CF_ = 3.2 Hz), 130.1 (2C), 129.3, 129.2 (*d*, *J*_CF_ = 2.7 Hz), 128.4 (*d*, *J*_CF_ = 3.5 Hz), 127.8, 127.7, 126.9 (*d*, *J*_CF_ = 8.9 Hz), 114.1 (2C), 92.5 (*d*, *J*_CF_ = 189.9 Hz), 84.4, 70.9 (*d*, *J*_CF_ = 28.3 Hz), 55.3, 27.8. ^19^F NMR (CDCl_3_, 188 MHz): δ −123.4. IR 2929, 1737, 1664, 1513, 1458, 1416, 1306, 1250, 1156, 1031 cm^−1^. ESI–MS: *m*/*z* 448.1 [*M* + H^+^].

***Synthesis of the title compound: cis*** **-4-fluoro-3-(4-meth­oxy­phen­yl)-1-oxo-2-phenyl-1,2,3,4-tetra­hydro­iso­quinoline-4-carb­oxy­lic acid, (**±**) 6:** 68.5 mg of **5** (0.20 mmol) were dissolved in CH_3_CN (1.2 ml), 85% (*w*/*w*) H_3_PO_4_ (86.5 µl, 0.80 mmol) was added and the mixture stirred at 323 K for 4 d. Afterwards, the mixture was extracted with CHCl_3_, dried over Na_2_SO_4_ and concentrated *in vacuo*. The crude product was purified by MPLC (EtOAc to EtOAc+0.1% FA) to isolate **(**±**) 6** as colourless crystals (yield 21.3 mg, 54.0 mmol, 36%; m.p. 461–462 K). ^1^H NMR (DMSO-*d*_6_, 400 MHz): δ 8.22–8.17 (*m*, 1H), 7.75–7.70 (*m*, 2H), 7.59–7.55 (*m*, 1H), 7.34–7.31 (*m*, 2H), 7.24–7.20 (*m*, 1H), 7.13–7.10 (*m*, 2H), 7.00–6.69 (*m*, 2H), 6.77–6.74 (*m*, 2H), 5.53 (*d*, *J* = 14.0 Hz, 1H), 3.66 (*s*, 3H). ^13^C NMR (DMSO-*d*_6_, 100 MHz): δ 167.6 (*d*, *J*_CF_ = 26.6 Hz), 161.6, 159.2, 140.8, 132.9 (*d*, *J*_CF_ = 1.5 Hz), 132.5 (*d*, *J*_CF_ = 19.3 Hz), 130.7 (*d*, *J*_CF_ = 3.0 Hz), 129.7, 129.2 (*d*, *J*_CF_ = 3.6 Hz), 128.8, 128.3, 127.5, 127.4, 127.0, 125.9 (*d*, *J*_CF_ = 6.6 Hz), 113.7 (2C), 92.6 (*d*, *J*_CF_ = 188.0 Hz), 68.7 (*d*, *J*_CF_ = 28.1 Hz), 55.0. ^19^F NMR (CD_3_OD, 188 MHz): δ −130.0. IR 2834, 2594, 1737, 1617, 1511, 1464, 1281, 1219, 1036 cm^−1^. ESI–MS: *m*/*z* 392.0 [*M* + H^+^].

## Refinement   

Crystal data, data collection and structure refinement details are summarized in Table 2 ▸. The H atoms were included in calculated positions and treated as riding: O—H = 0.84 Å, C–H = 0.95–1.00 Å with *U*_iso_(H) = 1.5*U*_eq_(O-hydroxyl,C-meth­yl) and 1.2*U*_eq_(C) for other H atoms.

## Supplementary Material

Crystal structure: contains datablock(s) I, global. DOI: 10.1107/S2056989017007186/su5371sup1.cif

Structure factors: contains datablock(s) I. DOI: 10.1107/S2056989017007186/su5371Isup2.hkl

CCDC reference: 1535140

Additional supporting information:  crystallographic information; 3D view; checkCIF report

## Figures and Tables

**Table 1:** Hydrogen-bond geometry (Å, °)

*D*—H⋯*A*	*D*—H	H⋯*A*	*D*⋯*A*	*D*—H⋯*A*
O14—H14⋯O11^i^	0.84	1.75	2.5645 (11)	163
C23—H23*C*⋯F12^ii^	0.98	2.50	3.2435 (14)	133

Symmetry codes: (i) 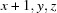; (ii) 
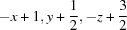
.

**Table 2:** Experimental details

Crystal data	
Chemical formula	C_23_H_18_FNO_4_
*M*_r_	391.38
Crystal system, space group	Monoclinic, *P*2_1_/*c*
Temperature (K)	100
*a*, *b*, *c* (Å)	8.4849 (11), 15.407 (3), 14.157 (2)
β (°)	102.598 (16)
*V* (Å^3^)	1806.1 (5)
*Z*	4
Radiation type	Mo *K*α
μ (mm^−1^)	0.11
Crystal size (mm)	0.28 × 0.20 × 0.18

Data collection	
Diffractometer	Bruker D8 Quest
Absorption correction	Multi-scan (*SADABS*; Bruker, 2014 ▸)
*T*_min_, *T*_max_	0.718, 0.746
No. of measured, independent and observed [*I* > 2σ(*I*)] reflections	48705, 3697, 3509
*R*_int_	0.024
(sin θ/λ)_max_ (Å^−1^)	0.625

Refinement	
*R*[*F* ^2^ > 2σ(*F* ^2^)], *wR*(*F* ^2^), *S*	0.032, 0.081, 1.05
No. of reflections	3697
No. of parameters	264
H-atom treatment	H-atom parameters constrained
Δρ_max_, Δρ_min_ (e Å^−3^)	0.38, −0.21

Computer programs: *APEX2* and *SAINT-Plus* (Bruker, 2014 ▸), *SHELXT* (Sheldrick, 2015*a* ▸), *SHELXL2014* (Sheldrick, 2015*b* ▸), *SHELXLE* (Hübschle *et al.*, 2011 ▸) and *Mercury* (Macrae *et al.*, 2008 ▸).

## supplementary crystallographic information

### Crystal data

**Table:**

C_23_H_18_FNO_4_	*F*(000) = 816
*M**_r_* = 391.38	*D*_x_ = 1.439 Mg m^−^^3^
Monoclinic, *P*2_1_/*c*	Mo *K*α radiation, λ = 0.71073 Å
*a* = 8.4849 (11) Å	Cell parameters from 9581 reflections
*b* = 15.407 (3) Å	θ = 2.8–28.3°
*c* = 14.157 (2) Å	µ = 0.11 mm^−^^1^
β = 102.598 (16)°	*T* = 100 K
*V* = 1806.1 (5) Å^3^	Block, colourless
*Z* = 4	0.28 × 0.20 × 0.18 mm

### Data collection

**Table:**

Bruker D8 Quest diffractometer	3697 independent reflections
Radiation source: microfocus sealed tube (Incoatec ImS)	3509 reflections with *I* > 2σ(*I*)
Multi-layer mirror monochromator	*R*_int_ = 0.024
Detector resolution: 10.24 pixels mm^-1^	θ_max_ = 26.4°, θ_min_ = 2.6°
φ and ω scans	*h* = −10→10
Absorption correction: multi-scan (SADABS; Bruker, 2014)	*k* = −19→19
*T*_min_ = 0.718, *T*_max_ = 0.746	*l* = −17→17
48705 measured reflections	

### Refinement

**Table:**

Refinement on *F*^2^	Secondary atom site location: difference Fourier map
Least-squares matrix: full	Hydrogen site location: inferred from neighbouring sites
*R*[*F*^2^ > 2σ(*F*^2^)] = 0.032	H-atom parameters constrained
*wR*(*F*^2^) = 0.081	*w* = 1/[σ^2^(*F*_o_^2^) + (0.0355*P*)^2^ + 1.0295*P*] where *P* = (*F*_o_^2^ + 2*F*_c_^2^)/3
*S* = 1.05	(Δ/σ)_max_ = 0.001
3697 reflections	Δρ_max_ = 0.38 e Å^−^^3^
264 parameters	Δρ_min_ = −0.21 e Å^−^^3^
0 restraints	Extinction correction: (SHELXL2016; Sheldrick, 2015b), Fc^*^=kFc[1+0.001xFc^2^λ^3^/sin(2θ)]^-1/4^
Primary atom site location: structure-invariant direct methods	Extinction coefficient: 0.0347 (17)

### Special details

**Table:**

Experimental. The crystal was immersed in a film of perfluoropolyether oil, mounted on a polyimide microloop (MicroMounts of MiTeGen) and transferred to stream of cold nitrogen (Bruker Kryoflex2).Melting points were determined on a Melting point meter MPM-H2 (Schorpp Geraetetechnik, Ueberlingen, Germany) and were not corrected. IR spectra were obtained using a JASCO FT/IR-6100 spectrometer (JASCO, Gross-Umstadt, Germany). TLC was performed on pre-coated aluminium sheets with silica gel 60 F_254_ (Macherey-Nagel, Dueren, Germany). ^1^H (400 MHz) and ^13^C (100 MHz) NMR spectra were recorded on a Bruker AV 400 instrument (Bruker Biospin, Ettlingen, Germany). ^19^F (188 MHz) NMR spectra were recorded on a Bruker Advance 200Hz Spektrometer (Bruker Biospin, Bremen, Germany) at 298 K. Chemical shifts are given in ppm and were calibrated on residual solvent peaks as internal standard (CDCl_3_: ^1^H 7.26 ppm, ^13^C 77.1 ppm; DMSO-d_6_: ^1^H 2.50 ppm, ^13^C 39.52 ppm; CD_3_OD: ^1^H 3.31 ppm, ^13^C 49.00 ppm (Gottlieb *et al.*, 1997)). NMR signals are specified as s (singlet), d (doublet), m (multiplet). Coupling constants *J* are given in Hz. Medium pressure liquid chromatography (MPLC) was performed on puriFlash^®^430 system (Interchim, Montluçon, France) using pre-packed silica gel 50 µ columns from Interchim (Montluçon, France). MS data were obtained using an Agilent 1100 Series LC/MSD Trap (Agilent Technologies, Boeblingen, Germany). Commercial available chemicals were used without further purification. Gottlieb, H. E., Kotlyar, V. & Nudelman, A. (1997). *J. Org. Chem.*62, 7512-7515.
Geometry. All esds (except the esd in the dihedral angle between two l.s. planes) are estimated using the full covariance matrix. The cell esds are taken into account individually in the estimation of esds in distances, angles and torsion angles; correlations between esds in cell parameters are only used when they are defined by crystal symmetry. An approximate (isotropic) treatment of cell esds is used for estimating esds involving l.s. planes.

### Fractional atomic coordinates and isotropic or equivalent isotropic displacement parameters (Å^2^)

**Table:**

	*x*	*y*	*z*	*U*_iso_*/*U*_eq_	
N1	0.33200 (10)	0.61320 (6)	0.66591 (6)	0.01065 (19)	
C2	0.24318 (12)	0.57637 (7)	0.72408 (7)	0.0111 (2)	
C3	0.32751 (12)	0.51757 (7)	0.80230 (7)	0.0111 (2)	
C4	0.24489 (13)	0.48959 (7)	0.87212 (8)	0.0137 (2)	
H4	0.139982	0.511308	0.871797	0.016*	
C5	0.31679 (13)	0.42986 (7)	0.94204 (8)	0.0159 (2)	
H5	0.262356	0.411839	0.990646	0.019*	
C8	0.48373 (12)	0.48743 (7)	0.80421 (7)	0.0110 (2)	
C7	0.55226 (13)	0.42559 (7)	0.87261 (8)	0.0146 (2)	
H7	0.656607	0.403150	0.872799	0.018*	
C6	0.46852 (14)	0.39665 (7)	0.94054 (8)	0.0167 (2)	
H6	0.515302	0.353782	0.986387	0.020*	
C9	0.56834 (12)	0.51784 (7)	0.72743 (7)	0.0104 (2)	
C10	0.50982 (12)	0.60708 (7)	0.68386 (7)	0.0103 (2)	
H10	0.538244	0.609658	0.618980	0.012*	
O11	0.09652 (9)	0.59098 (5)	0.71148 (6)	0.01599 (18)	
F12	0.53273 (7)	0.45825 (4)	0.64999 (4)	0.01453 (16)	
C13	0.75401 (12)	0.52245 (7)	0.76088 (8)	0.0113 (2)	
O14	0.82294 (9)	0.51358 (6)	0.68636 (6)	0.01713 (18)	
H14	0.919081	0.530769	0.701830	0.026*	
O15	0.82280 (9)	0.53632 (6)	0.84323 (6)	0.01743 (19)	
C16	0.59280 (12)	0.68403 (7)	0.74097 (7)	0.0108 (2)	
C17	0.55319 (13)	0.71191 (7)	0.82625 (8)	0.0133 (2)	
H17	0.469288	0.683326	0.848858	0.016*	
C18	0.63400 (13)	0.78083 (7)	0.87914 (8)	0.0142 (2)	
H18	0.606692	0.798420	0.937867	0.017*	
C19	0.75537 (12)	0.82394 (7)	0.84538 (8)	0.0125 (2)	
C20	0.79674 (13)	0.79631 (7)	0.76033 (8)	0.0139 (2)	
H20	0.880507	0.824902	0.737609	0.017*	
C21	0.71594 (13)	0.72720 (7)	0.70881 (8)	0.0131 (2)	
H21	0.744716	0.708909	0.650739	0.016*	
O22	0.83760 (9)	0.89465 (5)	0.88880 (6)	0.01602 (18)	
C23	0.81993 (14)	0.91544 (8)	0.98427 (8)	0.0175 (2)	
H23A	0.883876	0.967223	1.007349	0.026*	
H23B	0.857866	0.866633	1.027671	0.026*	
H23C	0.705912	0.926726	0.983278	0.026*	
C24	0.25598 (12)	0.67125 (7)	0.58933 (8)	0.0124 (2)	
C25	0.16993 (13)	0.74324 (7)	0.60909 (9)	0.0174 (2)	
H25	0.160006	0.754905	0.673429	0.021*	
C26	0.09829 (14)	0.79821 (8)	0.53368 (10)	0.0239 (3)	
H26	0.038507	0.847210	0.546762	0.029*	
C27	0.11357 (15)	0.78197 (8)	0.43966 (10)	0.0257 (3)	
H27	0.065221	0.819873	0.388597	0.031*	
C28	0.19975 (15)	0.71015 (9)	0.42085 (9)	0.0236 (3)	
H28	0.210460	0.698898	0.356566	0.028*	
C29	0.27096 (13)	0.65417 (8)	0.49529 (8)	0.0167 (2)	
H29	0.329296	0.604724	0.481866	0.020*	

### Atomic displacement parameters (Å^2^)

**Table:**

	*U*^11^	*U*^22^	*U*^33^	*U*^12^	*U*^13^	*U*^23^
N1	0.0066 (4)	0.0132 (4)	0.0116 (4)	0.0003 (3)	0.0007 (3)	0.0019 (3)
C2	0.0094 (5)	0.0113 (5)	0.0128 (5)	−0.0010 (4)	0.0025 (4)	−0.0019 (4)
C3	0.0101 (5)	0.0109 (5)	0.0119 (5)	−0.0024 (4)	0.0017 (4)	−0.0015 (4)
C4	0.0118 (5)	0.0154 (5)	0.0143 (5)	−0.0033 (4)	0.0039 (4)	−0.0023 (4)
C5	0.0180 (5)	0.0176 (5)	0.0127 (5)	−0.0070 (4)	0.0045 (4)	0.0002 (4)
C8	0.0101 (5)	0.0106 (5)	0.0121 (5)	−0.0027 (4)	0.0019 (4)	−0.0013 (4)
C7	0.0118 (5)	0.0131 (5)	0.0178 (5)	−0.0010 (4)	0.0009 (4)	0.0007 (4)
C6	0.0185 (5)	0.0149 (5)	0.0148 (5)	−0.0033 (4)	−0.0006 (4)	0.0040 (4)
C9	0.0088 (5)	0.0108 (5)	0.0111 (5)	−0.0006 (4)	0.0015 (4)	−0.0023 (4)
C10	0.0071 (5)	0.0133 (5)	0.0110 (5)	0.0001 (4)	0.0028 (4)	0.0014 (4)
O11	0.0079 (4)	0.0188 (4)	0.0215 (4)	0.0009 (3)	0.0038 (3)	0.0035 (3)
F12	0.0135 (3)	0.0146 (3)	0.0154 (3)	−0.0022 (2)	0.0030 (2)	−0.0051 (2)
C13	0.0096 (5)	0.0095 (5)	0.0150 (5)	0.0006 (4)	0.0034 (4)	0.0017 (4)
O14	0.0087 (4)	0.0271 (4)	0.0165 (4)	−0.0028 (3)	0.0047 (3)	−0.0019 (3)
O15	0.0109 (4)	0.0258 (4)	0.0146 (4)	−0.0015 (3)	0.0005 (3)	0.0002 (3)
C16	0.0090 (5)	0.0102 (5)	0.0126 (5)	0.0014 (4)	0.0010 (4)	0.0022 (4)
C17	0.0118 (5)	0.0135 (5)	0.0156 (5)	−0.0010 (4)	0.0052 (4)	0.0015 (4)
C18	0.0152 (5)	0.0144 (5)	0.0137 (5)	0.0004 (4)	0.0048 (4)	−0.0005 (4)
C19	0.0108 (5)	0.0102 (5)	0.0148 (5)	0.0002 (4)	−0.0009 (4)	0.0012 (4)
C20	0.0107 (5)	0.0148 (5)	0.0166 (5)	−0.0015 (4)	0.0040 (4)	0.0032 (4)
C21	0.0126 (5)	0.0148 (5)	0.0125 (5)	0.0008 (4)	0.0039 (4)	0.0016 (4)
O22	0.0173 (4)	0.0146 (4)	0.0162 (4)	−0.0053 (3)	0.0036 (3)	−0.0024 (3)
C23	0.0171 (5)	0.0191 (5)	0.0152 (5)	−0.0036 (4)	0.0015 (4)	−0.0036 (4)
C24	0.0078 (4)	0.0129 (5)	0.0150 (5)	−0.0030 (4)	−0.0009 (4)	0.0031 (4)
C25	0.0122 (5)	0.0151 (5)	0.0230 (6)	−0.0011 (4)	−0.0002 (4)	−0.0002 (4)
C26	0.0144 (5)	0.0142 (6)	0.0383 (7)	−0.0008 (4)	−0.0046 (5)	0.0042 (5)
C27	0.0196 (6)	0.0217 (6)	0.0287 (7)	−0.0075 (5)	−0.0102 (5)	0.0134 (5)
C28	0.0228 (6)	0.0285 (7)	0.0162 (6)	−0.0091 (5)	−0.0026 (5)	0.0069 (5)
C29	0.0152 (5)	0.0183 (6)	0.0157 (5)	−0.0032 (4)	0.0012 (4)	0.0021 (4)

### Geometric parameters (Å, º)

**Table:**

N1—C2	1.3560 (14)	C16—C21	1.3960 (15)
N1—C24	1.4444 (13)	C17—C18	1.3907 (15)
N1—C10	1.4774 (12)	C17—H17	0.9500
C2—O11	1.2386 (13)	C18—C19	1.3946 (15)
C2—C3	1.4878 (14)	C18—H18	0.9500
C3—C4	1.3987 (15)	C19—O22	1.3655 (13)
C3—C8	1.3992 (15)	C19—C20	1.3923 (15)
C4—C5	1.3912 (16)	C20—C21	1.3847 (15)
C4—H4	0.9500	C20—H20	0.9500
C5—C6	1.3899 (17)	C21—H21	0.9500
C5—H5	0.9500	O22—C23	1.4282 (13)
C8—C7	1.3920 (15)	C23—H23A	0.9800
C8—C9	1.5023 (14)	C23—H23B	0.9800
C7—C6	1.3880 (16)	C23—H23C	0.9800
C7—H7	0.9500	C24—C25	1.3895 (16)
C6—H6	0.9500	C24—C29	1.3899 (16)
C9—F12	1.4112 (12)	C25—C26	1.3940 (17)
C9—C10	1.5440 (14)	C25—H25	0.9500
C9—C13	1.5445 (14)	C26—C27	1.388 (2)
C10—C16	1.5187 (14)	C26—H26	0.9500
C10—H10	1.0000	C27—C28	1.384 (2)
C13—O15	1.2036 (14)	C27—H27	0.9500
C13—O14	1.3202 (13)	C28—C29	1.3935 (16)
O14—H14	0.8400	C28—H28	0.9500
C16—C17	1.3901 (15)	C29—H29	0.9500
			
C2—N1—C24	119.90 (8)	C21—C16—C10	119.43 (9)
C2—N1—C10	123.43 (9)	C16—C17—C18	121.29 (10)
C24—N1—C10	116.15 (8)	C16—C17—H17	119.4
O11—C2—N1	120.70 (10)	C18—C17—H17	119.4
O11—C2—C3	121.52 (9)	C17—C18—C19	119.57 (10)
N1—C2—C3	117.78 (9)	C17—C18—H18	120.2
C4—C3—C8	120.18 (10)	C19—C18—H18	120.2
C4—C3—C2	118.68 (9)	O22—C19—C20	115.65 (9)
C8—C3—C2	121.08 (9)	O22—C19—C18	124.66 (10)
C5—C4—C3	119.81 (10)	C20—C19—C18	119.67 (10)
C5—C4—H4	120.1	C21—C20—C19	120.09 (10)
C3—C4—H4	120.1	C21—C20—H20	120.0
C6—C5—C4	119.78 (10)	C19—C20—H20	120.0
C6—C5—H5	120.1	C20—C21—C16	120.96 (10)
C4—C5—H5	120.1	C20—C21—H21	119.5
C7—C8—C3	119.39 (10)	C16—C21—H21	119.5
C7—C8—C9	121.58 (9)	C19—O22—C23	117.14 (8)
C3—C8—C9	118.86 (9)	O22—C23—H23A	109.5
C6—C7—C8	120.17 (10)	O22—C23—H23B	109.5
C6—C7—H7	119.9	H23A—C23—H23B	109.5
C8—C7—H7	119.9	O22—C23—H23C	109.5
C7—C6—C5	120.53 (10)	H23A—C23—H23C	109.5
C7—C6—H6	119.7	H23B—C23—H23C	109.5
C5—C6—H6	119.7	C25—C24—C29	120.38 (10)
F12—C9—C8	107.70 (8)	C25—C24—N1	120.76 (10)
F12—C9—C10	105.86 (8)	C29—C24—N1	118.85 (10)
C8—C9—C10	113.83 (8)	C24—C25—C26	119.43 (11)
F12—C9—C13	107.42 (8)	C24—C25—H25	120.3
C8—C9—C13	114.13 (9)	C26—C25—H25	120.3
C10—C9—C13	107.41 (8)	C27—C26—C25	120.57 (12)
N1—C10—C16	112.29 (8)	C27—C26—H26	119.7
N1—C10—C9	110.66 (8)	C25—C26—H26	119.7
C16—C10—C9	114.28 (8)	C28—C27—C26	119.51 (11)
N1—C10—H10	106.3	C28—C27—H27	120.2
C16—C10—H10	106.3	C26—C27—H27	120.2
C9—C10—H10	106.3	C27—C28—C29	120.63 (12)
O15—C13—O14	125.92 (10)	C27—C28—H28	119.7
O15—C13—C9	123.50 (9)	C29—C28—H28	119.7
O14—C13—C9	110.50 (9)	C24—C29—C28	119.48 (11)
C13—O14—H14	109.5	C24—C29—H29	120.3
C17—C16—C21	118.42 (10)	C28—C29—H29	120.3
C17—C16—C10	122.12 (9)		
			
C24—N1—C2—O11	1.02 (15)	F12—C9—C13—O15	−148.60 (10)
C10—N1—C2—O11	172.43 (10)	C8—C9—C13—O15	−29.28 (14)
C24—N1—C2—C3	−179.57 (9)	C10—C9—C13—O15	97.91 (12)
C10—N1—C2—C3	−8.16 (15)	F12—C9—C13—O14	34.57 (11)
O11—C2—C3—C4	−10.34 (15)	C8—C9—C13—O14	153.90 (9)
N1—C2—C3—C4	170.26 (9)	C10—C9—C13—O14	−78.92 (10)
O11—C2—C3—C8	166.87 (10)	N1—C10—C16—C17	50.04 (13)
N1—C2—C3—C8	−12.53 (15)	C9—C10—C16—C17	−77.06 (12)
C8—C3—C4—C5	−1.71 (16)	N1—C10—C16—C21	−131.70 (10)
C2—C3—C4—C5	175.53 (10)	C9—C10—C16—C21	101.20 (11)
C3—C4—C5—C6	−1.68 (16)	C21—C16—C17—C18	−0.31 (16)
C4—C3—C8—C7	3.75 (15)	C10—C16—C17—C18	177.98 (9)
C2—C3—C8—C7	−173.41 (9)	C16—C17—C18—C19	1.09 (16)
C4—C3—C8—C9	179.22 (9)	C17—C18—C19—O22	176.94 (10)
C2—C3—C8—C9	2.05 (15)	C17—C18—C19—C20	−1.42 (16)
C3—C8—C7—C6	−2.42 (16)	O22—C19—C20—C21	−177.51 (9)
C9—C8—C7—C6	−177.76 (10)	C18—C19—C20—C21	0.99 (16)
C8—C7—C6—C5	−0.97 (17)	C19—C20—C21—C16	−0.21 (16)
C4—C5—C6—C7	3.03 (17)	C17—C16—C21—C20	−0.14 (15)
C7—C8—C9—F12	84.49 (12)	C10—C16—C21—C20	−178.47 (9)
C3—C8—C9—F12	−90.87 (11)	C20—C19—O22—C23	−169.09 (9)
C7—C8—C9—C10	−158.47 (9)	C18—C19—O22—C23	12.49 (15)
C3—C8—C9—C10	26.17 (13)	C2—N1—C24—C25	54.81 (14)
C7—C8—C9—C13	−34.68 (14)	C10—N1—C24—C25	−117.20 (11)
C3—C8—C9—C13	149.96 (9)	C2—N1—C24—C29	−125.51 (11)
C2—N1—C10—C16	−93.63 (11)	C10—N1—C24—C29	62.48 (12)
C24—N1—C10—C16	78.07 (11)	C29—C24—C25—C26	0.14 (16)
C2—N1—C10—C9	35.38 (13)	N1—C24—C25—C26	179.82 (10)
C24—N1—C10—C9	−152.92 (9)	C24—C25—C26—C27	−0.58 (17)
F12—C9—C10—N1	75.45 (10)	C25—C26—C27—C28	0.49 (18)
C8—C9—C10—N1	−42.65 (11)	C26—C27—C28—C29	0.05 (18)
C13—C9—C10—N1	−170.02 (8)	C25—C24—C29—C28	0.38 (16)
F12—C9—C10—C16	−156.62 (8)	N1—C24—C29—C28	−179.30 (10)
C8—C9—C10—C16	85.28 (11)	C27—C28—C29—C24	−0.48 (17)
C13—C9—C10—C16	−42.08 (11)		

### Hydrogen-bond geometry (Å, º)

**Table:**

*D*—H···*A*	*D*—H	H···*A*	*D*···*A*	*D*—H···*A*
O14—H14···O11^i^	0.84	1.75	2.5645 (11)	163
C23—H23*C*···F12^ii^	0.98	2.50	3.2435 (14)	133

Symmetry codes: (i) *x*+1, *y*, *z*; (ii) −*x*+1, *y*+1/2, −*z*+3/2.
